# A Review of Three Decades of Research Dedicated to Making Equine Bones Stronger: Implications for Horses and Humans

**DOI:** 10.3390/ani13050789

**Published:** 2023-02-22

**Authors:** Brian D. Nielsen

**Affiliations:** Department of Animal Science, Michigan State University, 474 S. Shaw Ln, East Lansing, MI 48824, USA; bdn@msu.edu

**Keywords:** equine, horse, bone, skeleton, exercise, injury, sprint, confinement, silicon, nutrition

## Abstract

**Simple Summary:**

Skeletal injuries are common in athletic horses. This literature review covers over three decades of research focused on preventing bone-related injuries and demonstrates how research develops over time. In an initial study evaluating the role dietary silicon can play in racehorse injuries, an observation of mineral loss from the cannon bone was observed after the commencement of training. Subsequent work revealed the loss was associated with horses being removed from pasture and placed into stalls, resulting in decreased mechanical loading on the skeleton. As bone responds to the load placed upon it, continued research focused on housing and exercise requirements to prevent such bone loss. Only short sprints are needed to maintain or increase bone strength. Conversely, endurance exercise, without high-speed exercise, fails to cause bone to become stronger. Exercise can be either forced or voluntary but having free access to exercise does not guarantee that animals will perform it. Thus, horse behavior needs to be taken into consideration. While proper nutrition is critical for bone health, it does not guarantee it without appropriate exercise. Pharmaceuticals impact various factors associated with bone health. Many items influencing equine bone health can also be applied to humans.

**Abstract:**

Much research has been conducted in an attempt to decrease skeletal injuries in athletic horses. The objective of this literature review is to compile the findings of over three decades of research in this area, make practical recommendations, and describe how research can develop over the years. An initial study investigating the role of bioavailable silicon in the diets of horses in race training produced the unexpected finding of decreased bone mineral content of the third metacarpus subsequent to the onset of training. Further studies revealed this decrease to be associated with stall housing eliminating high-speed exercise, leading to disuse osteopenia. Only relatively short sprints (between 50 and 82 m) were necessary to maintain bone strength and as few as one sprint per week provided the needed stimuli. Endurance exercise without speed fails to elicit the same benefits to bone. Proper nutrition is also required for optimal bone health, but without the right exercise, strong bone cannot be maintained. Several pharmaceuticals may have unintended consequences capable of impairing bone health. Many of the factors influencing bone health in horses also exist in humans including a sedentary lifestyle, improper nutrition, and pharmaceutical side-effects.

## 1. Introduction

For many decades, the high incidence rate of dorsal metacarpal disease (commonly known as bucked shins [1]) has been of concern to those in the horse racing industry, with 70% of two-year-old Thoroughbreds developing the problem, as reported by Norwood [2]. This ailment is similar to shin splints which has commonly plagued human athletes, and, in particular, runners [3]. In 1989, as an exercise rider of racing Thoroughbreds, the author often engaged in conversations with the trainer for whom he rode about the latest research into how to prevent bucked shins. At that time, there was limited research into how equine bone responds to training, with some of the most prominent work being presented by Dr. David Nunamaker of the New Bolton Center. His research provided some guidance and understanding on how training techniques affect this problem [4].

In October of 1990, during the running of the Breeders’ Cup Distaff, the catastrophic breakdown of the filly “Go For Wand” in front of the grandstands at Belmont Park in New York emphasized the need for greater research into preventing bone-related injuries [5]. Coincidentally, that same month, the author had commenced research for his graduate work at Texas A&M University focused on trying to find ways to strengthen bone and prevent skeletal injuries in performance horses. The following is a review of over 30 years of that research. Besides demonstrating how research evolves and how one project can lead to another, the three decades of research provides recommendations, supported by science, in how to decrease injuries in athletic horses, with implications for humans as well.

## 2. Bioavailable Silicon

The initial project began with the examination as to whether supplementing a bioavailable source of silicon (Si) could decrease injury rates in equine athletes [6]. The work was inspired by earlier reports on the essentiality of dietary Si and the implications for new bone development [7,8,9,10]. With 53 Quarter Horses in race training in the blinded and placebo-controlled study, benefits from supplementing sodium aluminosilicate (SZA), the Si source, were documented [6]. First, all three supplemented groups (low, medium, and high dosages) had more horses complete the required race program (nine races scheduled two weeks apart) than were injured compared to the control group which had more horses injured than were able to complete the study without injury. (It should be noted, injuries were not of catastrophic nature but simply were injuries that required the horses to miss days of training.) Further, the medium and high dosage groups completed substantially greater distances in training before experiencing an injury or completing the project if no injury occurred (90 and 83 km, respectively) compared to the control group (50 km). Finally, the medium treatment group had a faster average race time (20.3 s) than the control and low Si groups (20.7 s) at the race distance of 320 m. While supplementation was not believed to make horses faster, it was concluded that faster individuals within the medium treatment group were able to better withstand the rigors of race training without experiencing injury, contributing to the faster overall race time of that group compared to the control and low Si groups.

The specific mode of action for the benefits reported in that study could not be determined. Thus, several future studies used markers of bone turnover not readily available at the time of the initial study [6] to elicit possible reasons for the decrease in injury rates. Lang et al. found that supplemented yearling horses had decreased markers of bone resorption compared to unsupplemented controls [11]. Likewise, with broodmares, trends (*p* < 0.10) for altered bone resorption were observed in postpartum supplemented mares compared to controls [12]. Combined, the two studies by Lang and colleagues suggest that an altered rate of bone turnover may have influenced injury rates by allowing for a more rapid rate of bone repair that would prevent subclinical issues from becoming clinical. This was supported, to some degree, by work from Turner et al., who used 20 calves beginning at three days of age to test whether Si supplementation could impact their skeleton [13]. No differences in bone architecture or mechanical properties could be detected, but the rate of bone turnover again appeared to be altered. While the change in bone turnover could be deemed positive, there was an accompanying increase in the aluminum (Al) content of cortical bone and articular cartilage. The Si concentration was increased in the aorta, spleen, lung, muscle, and kidney of Si-supplemented calves, but Al was also increased in all tissues [14].

Only benefits from Si supplementation were noted in the prior studies with horses, but there were concerns about the amount of Al that was present in the SZA supplement (up to 130 g/kg). This prompted the evaluation of another bioavailable Si source without Al; that being oligomeric orthosilicic acid (OSA) [15]. Both SZA and OSA altered calcium (Ca) retention and boron metabolism compared to controls, but only OSA was able to alter Si retention, digestibility, and plasma concentration. Thus, it appeared to be a viable option to provide dietary bioavailable Si without adding substantial amounts of Al to the equine diet. Another option may be a mineral supplement from a marine source that was tested against limestone (to provide a similar amount of Ca). In that study, yearling horses given the marine mineral supplement had enhanced bone turnover. Perhaps not coincidentally, that mineral supplement also provided a similar amount of Si as did SZA, suggesting the benefits may have come from Si [16].

With the prospect of bioavailable Si aiding in bone health, products claiming to contain bioavailable Si became available commercially. While research had shown some benefits, anecdotally people made claims that providing sources of bioavailable Si also aided in resorption of certain joint lesions. As work by Reynolds and colleagues had previously documented spontaneous resorption of lesions without any outside intervention in young, growing horses [17], skepticism accompanied these claims. To investigate this, 44 two-year-old Standardbreds were radiographed to identify osteochondrotic defects in the fetlock and hock joints [18]. From that group, eight horses met the inclusion criteria and were pair-matched and assigned to a control group (receiving a placebo) or a treatment group (receiving 200 g of a bioavailable Si source). Horses were kept on their respective treatment for 120 days at which point they were radiographed again. No treatment differences were observed, though it was acknowledged that the number of horses completing the project was small, limiting the ability to conclusively say treatment had no effect. However, no findings in the study supported the anecdotal reports of lesion regression with supplementation. It was acknowledged that supplementation may need to occur at a younger age to prevent the development of osteochondrosis.

The concern regarding age of horse when supplementing was echoed by Pritchard et al. Retired Standardbred racehorses were supplemented with a source of bioavailable Si to examine whether it could affect lameness, particularly through its potential role in collagen synthesis [19]. In this 84-day study, 10 horses were pair-matched and assigned to a Si-supplemented group or control. No treatment differences in lameness examination scores, radiographic scores of joints, or other indices of collagen degradation or synthesis were observed. With the mean age of horses being over 10 years, this study questioned whether supplementation provides little benefit in the aged animal. It also questioned whether the dosage provided in a commercially available form (0.3 g supplement/100 kg BW per d) was insufficient to elicit a response. Pritchard et al. also examined supplementation in broilers supplemented from day 1 after hatching until 42 days of age [20]. No differences were seen in bone density, morphology, and strength measures between treatments, though supplementation altered serum mineral concentrations. Like with the previous Standardbred study [19], the dosage recommended by the manufacturer was below the intakes previously utilized in studies that reported positive influences on bone. Combined, these studies suggest the importance of careful scrutiny of commercial products that make claims based on studies that were not performed on their products, or that recommend dosages not shown to produce benefits in published research.

## 3. Bone Loss in Early Training Associated with Stall-Housing

In an attempt to find a dietary supplement that can prevent skeletal injuries, arguably a more important finding occurred by happenstance. In the initial study utilizing 53 horses in race training, radiographs were taken of the third metacarpus throughout the study. This was done to examine differences in bone mineral content using radiographic photodensitometry measured in radiographic bone Al equivalences (RBAE) [21]. This technique allows the optical density of the various steps of an Al stepwedge penetrometer attached to the radiographic cassette to be equated to the peak optical density of each cortex of the third metacarpal (reported in mm Al) [22]. The bone optical density is influenced by both the thickness of the bone and the density of the bone. This technique was later refined to allow the total bone mineral content of a cross-section of the third metacarpus to be assessed [23]. While not as precise as techniques such as computed tomography [24], which has been shown to be strongly correlated to bone ash weight [25], it is a non-invasive technique that has the advantage of not requiring sedation and being relatively inexpensive to use. In recent years, this technique has been modified for use with digital radiographs [26] and, when used with digital radiographs, the need to use unprocessed images has been recognized [27]. 

**Figure 1:**
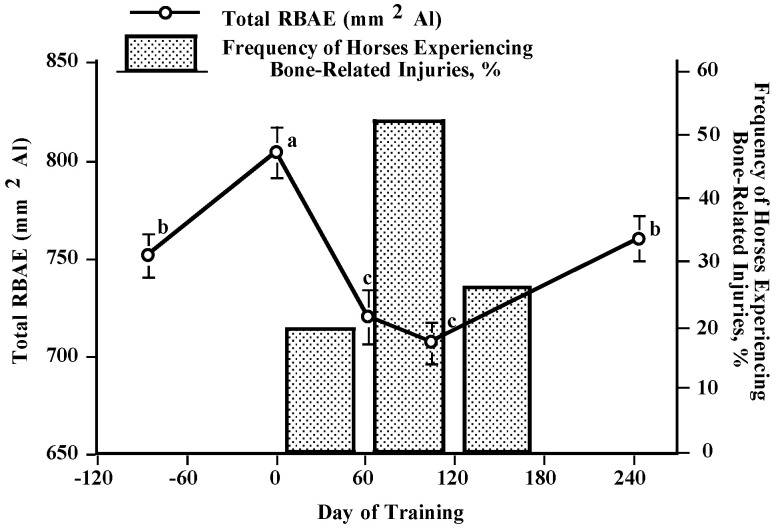
Total radiographic bone Al equivalences (RBAEs) of all horses and periods during which bone-related injuries occurred [21]. ^a,b,c^ Days lacking a common superscript differ (*p* < 0.05).

Using this technique, the initial racehorse study revealed a surprising result. When horses commenced race training, the RBAE (again, an estimate of bone mineral content) had decreased by day 62 of training (Figure 1) [21]. The RBAE remained low through day 104 of the study but had begun to increase by the conclusion of the study at day 244. These findings were surprising as most physiological systems are typically believed to increase in strength when athletic training commences (cardiovascular, muscular, respiratory). Thus, to find the skeletal system losing bone mass was confusing and alarming. Further, it should be noted that most bone-related injuries happened between days 60 and 120 of training when the bone mass was at its lowest. Accompanying this, horses began racing during week 9 of the study. While it was surprising that horses were losing bone mass during the initial stages of training, it was not surprising that the greatest injury rates were occurring at the time when bone was the weakest and horses were beginning to race. The combination of fast speeds and weak bones logically results in injuries.

At the time of the study, most discussions about modifications of equine bone focused on bone remodeling—the process by which old or damaged bone is replaced by new bone [28]. In theory, the loss of bone at the start of training could be explained if equine bone was not sufficiently strong to withstand the rigors of training resulting in damaged bone that needed to be removed to allow new bone to replace it. If that were to occur, it raised the question as to whether the amount of dietary mineral recommended by the 1989 Horse NRC [29] would be sufficient once new bone began to be deposited. Given that shin soreness in humans had been linked with low Ca intake [30], there was concern that insufficient Ca in the diet might leave horses susceptible to bone-related injuries.

To address this, 10 previously untrained Quarter Horses were put into race training for 112 days [31]. They were fed diets balanced to meet NRC requirements [29]. Radiographs revealed a decrease in bone mineral content of the third metacarpus by day 56 of training, prior to the initiation of speedwork being introduced during the second half of the study. This decrease in bone mass was similar to the decrease seen in the prior study using 53 horses. In the second half of the study, bone mass increased, and was accompanied by greater Ca retention. This inspired a follow-up study using 12 previously untrained horses, divided into one group that received Ca and P similar to the NRC recommended concentrations [29] and another group that received them at higher concentrations [32]. Of note, in the study utilizing only 10 horses, there was an adaptation period of 19 days in which horses were walked on a mechanical walker for one hour per day. In contrast, in the later study using 12 horses, the adaptation period was decreased to 9 days and horses were walked on a mechanical walker twice a day. This was done to minimize potential changes associated with a decrease in load being placed on the skeletal system due to stall confinement prior to training as there was increasing evidence that restriction of activity could be associated with loss of bone mass [33].

While the increased allocation of dietary mineral did result in increased Ca retention for young horses in training, another observation was that the changes in RBAE of the third metacarpal did not appear as great in the study with the shortened adaptation period [32] as they did in the study with the longer adaptation period [31].

Reflecting on the initial study in which bone loss had been observed in the 53 horses in race training [21], it was noted that prior to entering race training, the young horses had been moved from pasture housing and placed into box stalls. By eliminating access to free exercise and having no fast exercise during the early part of training, it was hypothesized that the loss of bone may have been caused by the lack of loading on the skeleton. At the time, it was not commonly believed among horsemen that confinement had anything to do with bone loss. Fortuitously, one of the researchers in the latter two studies [31,32] had no previous horse experience that could bias her, though she had extensive experience with bone loss in human bed-rest patients. When the idea was presented to her, she responded that it was logical. Lacking horse experience kept her from having any preconceived notions based upon how things have traditionally been done with horses.

To test the hypothesis that stalling of horses with no access to high-speed exercise was responsible for bone loss, 16 Arabian yearlings, previously housed together on pasture, were randomly divided into two groups [34]. Half of the horses remained on pasture while the other half were moved into box-stall housing with one hour of walking daily on a mechanical walker. By day 28 of the study, the RBAE of the third metacarpus of box-stalled horses had decreased and remained low throughout the 140-day study. Even when horses were started under saddle and began race training after 12 weeks on the study, no increase in bone mass was seen in the stalled horses during 8 weeks of slow racetrack training without speed. The concentration of serum osteocalcin (a marker of bone formation) was lower and urinary deoxypyridinoline (a marker of bone resorption) was higher in the confined horses at days 14 and 28, respectively, compared with the pastured horses, and both markers subsequently returned to baseline in the confined horses. Those alterations suggest bone formation decreased and bone resorption increased in the confined horses, thus, explaining the loss of bone. With this study closely mirroring how yearling racehorses are managed while being prepared for sales and during the early training, it was concerning to note that horses kept in stalls had lower bone mass at the end of the nearly five-month study than they did when they started it. The results suggested a probable cause for the high injury rates observed in young horses managed in that fashion.

At the same time, a study examined bone mineral content of the third metacarpus in 11 mature Arabian horses (ages 4 to 7 years) that had been previously conditioned but were then placed into box stalls for 12 weeks [35]. Despite being walked on a mechanical walker daily in two 30 min exercise bouts, and being fed dietary Ca at twice the 1997 NRC-recommended amounts [29], horses lost bone mass.

While those studies showed that having horses housed in box stalls without access to high-speed exercise (either forced or voluntary if housed on pasture) resulted in bone loss, it raised the question as to whether having horses housed on pasture completely (as opposed to partially) was necessary to avoid bone loss. To test this, 17 weanling Arabian horses were randomly assigned to three treatment groups: (1) housed on pasture, (2) housed in stalls, and (3) housed in stalls for 12 h per day and housed on pasture for 12 h per day [36]. After 56 days, greater increases in bone mass of the third metacarpal were observed in both groups allowed access to pasture, as opposed to the group confined completely to stalls. Thus, it appeared that even partial turnout could prevent bone loss associated with disuse.

At the end of that study, all weanlings were returned to pasture housing. Close to a year later, horses were radiographed again as part of a follow-up study [37]. Being returned to pasture allowed the horses subjected to complete stalling to have a similar bone mineral content of their third metacarpus as did their counterparts that had pasture housing. The results of that study suggest that short-term stall housing of young horses does not doom them to lower bone mass throughout life, assuming the return to pasture occurs while the horses are young and experiencing relatively fast bone growth. However, this study did not answer whether the same holds true for mature horses. A later study that included mature horses was able to detect alterations in bone metabolism markers indicative of enhanced bone formation when stalled horses were returned to pasture after 4 weeks though it is unclear if these alterations would result in fully restoring any bone lost due to stalling [38].

## 4. Role of Exercise in Bone Development

While it was assumed that the differences in bone associated with pasture- versus stall-housing were due to lack of high-speed exercise experienced by the stalled horses, other factors could have played a role including such things as differences in nutrition or exposure to sunlight. To determine if stalling caused disuse osteopenia (the loss of bone associated with lack of mechanical loading), exercise needed to be the sole factor that was altered in a research study. To test this, 18 juvenile bull calves were used to allow for a terminal study in which actual bone strength could be tested, as opposed to only using an indirect measure of bone mineral content [39]. They were assigned to one of three treatment groups: (1) group-housed, (2) confined with no exercise, and (3) confined with exercise consisting of running 50 m on a concrete surface once daily, 5 d/week for the duration of the six-week study. At the conclusion of the study, calves were humanely sacrificed, and the third and fourth metacarpal bone was scanned using computed tomography to determine cross-sectional geometry and bone mineral density. The bones were then subjected to a three-point bending test to failure. Exercised calves had increased cortical thickness and decreased medullary cavity area, as well as increased cortical bone density. There was also a trend for higher fracture force compared to the confined calves. The changes were considered quite remarkable given they were the result of running a cumulative distance of only 1500 m during the six-week study.

Despite the obvious benefit such a small amount of sprinting produced on bone, it was recognized that individuals in the horse industry might be reluctant to believe the results, obtained with calves, could be applicable to horses. Thus, we also performed a study utilizing 18 weanlings divided into three groups: (1) group-housed, (2) box stall-confined with no exercise, and (3) box stall-confined with a daily sprint of 82 m, 5 d/week for the duration of the 8-week study [40]. Using the RBAE technique [22,23], increased bone mineral content and altered bone geometry were noted in the confined horses that were allowed to sprint short distances (a cumulative distance of 3280 m over 8 weeks), compared to the confined horses that experienced no sprinting.

Exercise also produced beneficial changes in exercising swine [41]. Gestating gilts were divided into treatment groups receiving (1) no exercise, (2) low exercise (122 m/d, 5 d/week), or (3) high exercise (122 m two d/week and 427 m three d/week). All animals were stall housed during gestation and the study took place between d 35 and 110 of gestation. Bone density and breaking force were greater in the exercised gilts compared to the gilts receiving no exercise, with the additional benefits of exercise being shown in piglet survivability.

While both of those studies evaluated the response of bone to exercise performed five days per week [39,40,41], the question remained as to whether that frequency was needed, or whether fewer times per week would result in the same benefits. To examine this, 24 juvenile bull calves were divided into four groups: a control group receiving no exercise, or groups receiving a 71 m sprint either once, three times, or five times per week for the duration of the six-week study [42]. Calves were humanely euthanized and the left fused third and fourth metacarpal bones were scanned using computed tomography and tested for fracture force using four-point bending. All exercised groups had greater dorsal cortical widths compared to control animals and the fracture force was greater for all exercised groups than the control group. There were no differences between exercised groups, indicating that only one short sprint per week was required to enhance bone strength. With one 71 m sprint per week conducted over six weeks, the cumulative distance sprinted in a month and a half was 426 m, resulting in over a 20% increase in bone strength compared to calves that received no sprinting. This dramatic difference, brought about by such a small amount of high-speed exercise, should be alarming to those in the horse industry that do not afford any opportunity for their horses to run at speed and, are thus, developing weakened bone.

## 5. Speed (Load) versus Strides (Cycles)

It should be noted that not all exercise produces similar benefits. As bone responds to mechanical loading, any alterations in how strides are taken can impact bone formation. For instance, circular exercise such as lunging is commonly done with horses. While believed to be detrimental to joint health, various bone parameters have been shown to be altered between inside and outside legs of juvenile bull calves exercised in only one direction five days per week in a study lasting seven weeks [43].

Further, bone responds more to mechanical strain (the amount of bending) than it does the number of times it is bent [44]. This means the force which is exerted on the bone has more influence than does the number of cycles of bending or, for instance, strides the animal takes. In rats, just 36 cycles of bending three times per week was sufficient to prevent disuse osteopenia associated with immobilization [45]. In an avian model, it was determined that only four consecutive cycles prevented bone loss, and 36 cycles increased new bone formation [46]. Increasing the number of cycles from 36 to 1800 resulted in no additional strengthening of bone.

With bone responding to the magnitude of strain (how much it bends) as opposed to how often it bends, it was not surprising that, during a conditioning study lasting 78 days, carrying weight (progressively increasing the amount of weight carried up to 45 kg) resulted in increased RBAE in young horses exercised in a walker compared to control horses that were exercised similarly, but without carrying supplemental weight [47]. Prior to entering the conditioning period, all horses were stall-confined for 108 days, during which time they had lost bone mineral content of the third metacarpal, also reaffirming the detrimental effects that stalling without access to proper exercise has on bone.

Likewise, while it has long been believed that months of slow training will increase bone strength and prevent injuries [48], science does not support this. Using 11 two-year-old Arabians that were split into two groups, the influence endurance exercise has on bone mineral content of the third metacarpus was examined [49]. One group was trained on a high-speed treadmill for 90 days, with training consisting of walking (1.6 m/s), trotting (4 m/s), and cantering (8 m/s) at increasing distances until the target of 60 km/d was met. Starting on day 90, the exercised horses were placed on a regular exercise schedule including a 60 km endurance test every three weeks. The other group served as controls and lived on pasture without any forced exercise. Radiographs of the third metacarpal were taken at the start of the study and at day 162. No differences were seen between treatments in RBAE, suggesting endurance exercise does little to alter bone optical density compared with free-choice exercise on pasture. However, it would be interesting to repeat this study with greater numbers. The total RBAE of the endurance-exercised group was 493 ± 48 mm Al^2^ at the start of the study and finished at 424 ± 44 mm Al^2^. The total RBAE of the control group (pasture-housed with no forced exercise) started at 394 ± 48 mm Al^2^ and finished at 429 ± 48 mm Al^2^. In previous studies [39,40], it was noted that although animals had access to exercise because they were group-housed, it did not guarantee they did any sprinting. In the endurance study [49], it is likely that after the horses had their daily endurance training bouts and were returned to a group-housed setting, they likely opted to eat, drink, and rest as opposed to doing any additional fast exercise. By contrast, those in the control group likely occasionally played, including sprinting, which would have a greater impact on bone strength than would the endurance exercise.

Similarly, no treatment differences in RBAE and biomarkers of cartilage turnover were reported between yearling horses conditioned at a walk on an aquatic treadmill, on a dry treadmill, and those receiving no forced exercise [50]. However, all horses had access to turnout for about 10 h per day where voluntary exercise was allowed, and it was believed that this access to turnout had a greater effect on bone and cartilage than did the forced exercise at a slow speed.

Voluntary activity can play an important role in maintaining or increasing bone strength if the opportunity is allowed and if the animal takes advantage of such. Given that some of the previous studies have suggested group-housed animals allowed free access to exercise (as opposed to confinement-housed animals not allowed to exercise at speed) do not always take advantage of this opportunity, it is critical for horse caretakers to take horse behavior into consideration. In a recent study, the impact of weather was investigated on activity of horses while on pasture [51]. An interesting finding was a difference in activity between horses housed on farms located 3.5 km apart. That suggested that factors other than weather played an important role. The farm reporting greatest activity had horses primarily bred for racing. However, one of the horses in that group was a 33-year-old Spotted American Saddle Horse with pituitary pars intermedia dysfunction. Despite her age, disease status, and breed, it is likely her increased activity was due to being kept with horses bred for racing and who often ran. When they ran, she did also. The desire to be with other horses is strong and has been reported before [52]. This can increase the frequency of sprinting if kept with other horses inclined to sprint or can decrease the frequency if kept with horses not inclined to do so.

Thus, housing horses on pasture does not guarantee they will perform exercise necessary to enhance bone strength, but it does increase the likelihood of it. By contrast, if confined to a stall and never afforded the opportunity to run, it can be assured that skeletal strength will be compromised.

## 6. Pharmaceutical Factors

Besides nutritional and biomechanical influences on bone, pharmaceutical influences also exist, some with unintended consequences. In recent years, there has been growing concern regarding the use of bisphosphonates. Approved in 2014 to treat navicular disease in horses over the age of four years, bisphosphonates have also been used extra-label for other skeletal issues. With bisphosphonates inhibiting osteoclasts, whose function is to resorb bone, there is concern regarding whether this will impair bone healing, leaving horses, particularly young horses in training, more susceptible to injury [53]. In humans, long-term use has been linked to increased risks of some types of fractures.

Furosemide is commonly given to racehorses in North America in an attempt to decrease the incidence of exercise-induced pulmonary hemorrhage. As the usage of such has been shown to negatively impact Ca balance for several days after administration, concern existed as to whether it could have detrimental effects on bone if this effect persisted beyond that. Using a crossover design with ten horses, serving as both controls and furosemide-treated, during two 8-day periods of total collection of urine and feces, it was shown that Ca balance returned to baseline in three days after furosemide administration and does not present much risk to bone, particularly since there seemed to be a compensatory effect on of having lower fecal Ca loss in treated horses by the end of the collection period [54].

Likewise, omeprazole has been commonly provided to aid in healing or preventing gastric ulcers in horses, but concerns have existed whether the suppression of gastric acid may inhibit absorption of Ca and thus impair skeletal health. Using a preventative dose (1 mg/kg BW daily) for up to two months provided no indication of such [55]. Usage for longer periods or at the treatment dose (4 mg/kg BW daily) could still pose a risk. Incidental findings of alterations in markers of bone formation, likely associated with the stalling of horses near the end of the study, reaffirm the concern with stalling of horses. In particular, many athletic horses are stalled, and these would often be the same horses that have a greater propensity to develop ulcers. These findings reaffirm the concern surrounding the stalling of horses without access to exercise capable of supporting optimal skeletal health.

## 7. Conclusions

Many without research experience believe solutions to problems can often be achieved through a single study. By contrast, this paper highlights how it can sometimes take decades, utilizing dozens of studies, to be able to make solid, research-based recommendations. Often a single study will inspire other studies to answer new questions that arise. Additionally, sometimes research provides incidental findings that may prove to be more important than are the findings for which the study was designed.

While originally looking for a nutritional approach to preventing skeletal injuries, a loss of bone mass of the third metacarpal of horses in race training was shown. Trying to find ways to prevent that bone loss led to the realization that the loss was due to stalling of horses without access to speed. Future studies showed that pasture turnout prevented that bone loss and as little as one short sprint per week made dramatic differences in bone strength. Failure to provide athletic horses with such an opportunity would seemingly put them at increased risk of injury—particularly once high-speed work resumes.

Exploring whether stalling of horses caused the loss of bone mass was inspired by a mentor who had no horse experience and, thus, was not biased by how things have traditionally been done in the horse industry. Currently, many in the horse industry believe that training of young, growing horses is detrimental and should not be done. However, scientists working in this area realize that is not the case [56]. Bone modeling, the process by which changes in the size, shape, and strength of bone occur, happens primarily in the juvenile animal. Once skeletally mature, changes primarily occur through bone remodeling—the process which involves simply replacing old or damaged bone. Thus, little change in size, shape, and strength can occur once skeletal maturation is complete.

Nutrition does play an important role in bone health. Having the necessary nutrients in proper balance is crucial to bone development, particularly in the growing horse [57]. However, examining studies using markers of bone metabolism to detect treatment differences, those studies involving changes in nutrition usually failed to show differences, whereas studies involving altering exercise usually resulted in significant differences in those biochemical markers [58]. Likewise, in young female humans, only childhood physical activity had a significant positive on bone density, as opposed to nutritional and other lifestyle factors that had no influence [59]. Granted, this is based upon the assumption of adequate Ca intake and other nutrients, and this is especially true after menopause [60]. These studies emphasize the benefit of an active lifestyle on the skeletal system, particularly when young and growing to achieve a higher peak bone mass upon maturity. However, activity is still crucial when older as a sedentary lifestyle hastens bone loss and is permissive to osteoporosis, regardless of diet. These findings do not lessen the importance of proper nutrition on bone health, it simply suggests that once requirements are met, providing additional nutrients cannot assure good bone health. It also suggests it is not possible to have optimal bone health if no exercise, or the wrong type of exercise, is provided, regardless of what is consumed through the diet. While the temptation by some is to fault nutrition for skeletal injuries, more often it is improper training, management, or lifestyle that is to blame.

Research also suggests caution be taken when providing pharmaceuticals that may inhibit mineral absorption or enhance mineral loss, though proper exercise and nutrition may mitigate potential negative effects. More concerning are pharmaceuticals that inhibit normal bone metabolism, such as by reducing osteoclastic activity, as this can impair normal bone healing [53]. Additionally, potential analgesic effects should be of great concern whether with pharmaceuticals such as bisphosphonates or corticosteroids [61]. Masking pain when an injury is still present increases the chance for greater injury, potentially even catastrophic in nature, to occur.

Though the intent of much of the afore-mentioned research was to improve the lives, health, and well-being of horses, implications also exist for humans. A sedentary lifestyle, without sufficient mechanical loading of bone, will lead to a weakened skeleton. With the lifespan of humans being longer than horses, failure to strengthen the skeleton when young, and failure to maintain skeletal strength when mature, likely will increase the risk of skeletal injury throughout life and increase the chance of developing osteoporosis when older. Improper nutrition will increase the risk, though proper nutrition, without the correct form of exercise, will not prevent it. Fortunately, as with horses, the proper exercise does not require long bouts of exercise. Short bouts (only about 20 cycles) of bone-centric exercise can increase bone strength without the damage that repeated cycles can cause [62]. Again, like with horses, having a skeleton adapted for high load requires loading while young, continuing through maturity. Attempts to improve skeletal health once mature is more difficult to accomplish and much greater care needs to be given to managing loads placed upon it and preventing bone loss when aging.

With both horses and humans, many of the skeletal injuries that develop are the result of bone being ill-prepared for high loads due to sedentary periods without loading. As quoted by Dr. Gary D. Potter, the author’s major professor in graduate school at Texas A&M University, when an old horse trainer was asked what he believed to be the major cause of injuries in his racehorses, his answer was respiratory problems. The trainer claimed he did not know why, but it seemed like every time one of his horses became sick and needed to be rested for a while, it became injured when he put it back in training. With decades of research to support it, the answer is clear.

## Data Availability

Not applicable.

## References

[1] Couch S., Nielsen B. (2017). A Review of Dorsal Metacarpal Disease (Bucked Shins) in the Flat Racing Horse: Prevalence, Diagnosis, Pathogenesis, and Associated Factors. J. Dairy Vet. Anim. Res..

[2] Norwood G.L. (1978). The Bucked-Shin Complex in Thoroughbreds. Proc. 24th Annu. Conv. Am. Assoc. Equine Pract..

[3] Patil S.S.D. (2016). Shin Splints. Foot and Ankle Sports Orthopaedics.

[4] Nunamaker D.M., Butterweck D.M., Provost M.T. (1990). Fatigue Fractures in Thoroughbred Racehorses: Relationships with Age, Peak Bone Strain, and Training. J. Orthop. Res..

[5] Nielsen B.D. (1996). Mineral Balance and Physiological Responses Associated with the Bone Modeling/Remodeling Process in Young Racehorses at the Onset of Training.

[6] Nielsen B.D., Potter G.D., Morris E.L., Odom T.W., Senor D.M., Reynolds J.A., Smith W.B., Martin M.T., Bird E.H. (1993). Training Distance to Failure in Young Racing Quarter Horses Fed Sodium Zeolite A. J. Equine Vet. Sci..

[7] Carlisle E.M. (1974). Proceedings: Silicon as an Essential Element. Fed. Proc..

[8] Carlisle E.M. (1980). A Silicon Requirement for Normal Skull Formation in Chicks. J. Nutr..

[9] Carlisle E.M. (1980). Biochemical and Morphological Changes Associated with Long Bones Abnormalities in Silicon Deficiency. J. Nutr..

[10] Carlisle E.M. (1982). The Nutritional Essentiality of Silicon. Nutr. Rev..

[11] Lang K.J., Nielsen B.D., Waite K.L., Hill G.A., Orth M.W. (2001). Increased Plasma Silicon Concentrations and Altered Bone Resorption in Response to Sodium Zeolite a Supplementation in Yearling Horses. J. Equine Vet. Sci..

[12] Lang K.J., Nielsen B.D., Waite K.L., Hill G.M., Orth M.W. (2001). Supplemental Silicon Increases Plasma and Milk Silicon Concentrations in Horses. J. Anim. Sci..

[13] Turner K.K., Nielsen B.D., O’Connor-Robison C.I., Rosenstein D.S., Marks B.P., Nielsen F.H., Orth M.W. (2008). Sodium Zeolite A Supplementation and Its Impact on the Skeleton of Dairy Calves. Biol. Trace Elem. Res..

[14] Turner K.K., Nielsen B.D., O’Connor-Robison C.I., Nielsen F.H., Orth M.W. (2008). Tissue Response to a Supplement High in Aluminum and Silicon. Biol. Trace Elem. Res..

[15] O’Connor C.I., Nielsen B.D., Woodward A.D., Spooner H.S., Ventura B.A., Turner K.K. (2008). Mineral Balance in Horses Fed Two Supplemental Silicon Sources. J. Anim. Physiol. Anim. Nutr..

[16] Nielsen B.D., Cate R.E., O’Connor-Robison C.I. (2010). A Marine Mineral Supplement Alters Markers of Bone Metabolism in Yearling Arabians. J. Equine Vet. Sci..

[17] Reynolds J.A., Morris E.L., Senor D.M., Frey K.S., Reagan D., Weir V.A., Elslander J., Potter G.D. (1992). The Incidence of Bone Lesions in the Carpal and Tarsal Regions and the Rate of Physeal Closure in Weanling Quarter Horses. J. Equine Vet. Sci..

[18] Turner K.K., Nielsen B.D., O’Connor C.I., Rosenstein D.S. (2007). Silicon Supplementation and Osteochondrotic Lesions in 2-Year-Old Standardbreds: A Preliminary Study. Equine Comp. Exerc. Physiol..

[19] Pritchard A., Nielsen B.D., Robison C., Manfredi J.M. (2020). Low Dietary Silicon Supplementation May Not Affect Bone and Cartilage in Mature, Sedentary Horses. J. Anim. Sci..

[20] Pritchard A., Robison C., Nguyen T., Nielsen B.D. (2020). Silicon Supplementation Affects Mineral Metabolism but Not Bone Density or Strength in Male Broilers. PLoS ONE.

[21] Nielsen B.D., Potter G.D., Morris E.L., Odom T.W., Senor D.M., Reynolds J.A., Smith W.B., Martin M.T. (1997). Changes in the Third Metacarpal Bone and Frequency of Bone Injuries in Young Quarter Horses during Race Training—Observations and Theoretical Considerations. J. Equine Vet. Sci..

[22] Meakim D.W., Ott E.A., Asquith R.L., Feaster J.P. (1981). Estimation of Mineral Content of the Equine Third Metacarpal by Radiographic Photometry. J. Anim. Sci.

[23] Nielsen B.D., Potter G.D. Accounting for Volumetric Differences in Estimates of Bone Mineral Content from Radiographic Densitometry. Proceedings of the 15th Equine Nutrition and Physiology Symposium.

[24] Waite K.L., Nielsen B.D., Rosenstein D.S. (2000). Computed Tomography as a Method of Estimating Bone Mineral Content in Horses. J. Equine Vet. Sci..

[25] Pritchard A., Robison C., Nielsen B.D. (2020). Research Note: Bone Ash from Immature Broilers Correlates to Bone Mineral Content Calculated from Quantitative Computed Tomography Scans. Poult. Sci..

[26] O’Connor-Robison C.I., Nielsen B.D. (2013). Comparison of Two Software Packages for Determining Radiographic Bone Aluminum Equivalent Values. Comp. Exerc. Physiol..

[27] Emmert B.J., Robison C.I., Pritchard A., Nielsen B.D. (2022). Comparison of Bone Mineral Content of the Equine Third Metacarpal to Total Radiographic Bone Aluminum Equivalents From Unprocessed Digital Radiographs. J. Equine Vet. Sci..

[28] Heaney R.P. (1994). The Bone-Remodeling Transient: Implications for the Interpretation of Clinical Studies of Bone Mass Change. J. Bone Miner. Res..

[29] (1989). Nutrient Requirements of Horses.

[30] Myburgh K.H., Grobler N., Noakes T.D. (1988). Factors Associated With Shin Soreness in Athletes. Phys. Sport..

[31] Nielsen B.D., Potter G.D., Greene L.W., Morris E.L., Murray-Gerzik M., Smith W.B., Martin M.T. (1998). Characterization of Changes Related to Mineral Balance and Bone Metabolism in the Young Racing Quarter Horse. J. Equine Vet. Sci..

[32] Nielsen B.D., Potter G.D., Greene L.W., Morris E.L., Murray-Gerzik M., Smith W.B., Martin M.T. (1998). Response of Young Horses in Training to Varying Concentrations of Dietary Calcium and Phosphorus. J. Equine Vet. Sci..

[33] Knowles T.G., Broom D.M. (1990). Limb Bone Strength and Movement in Laying Hens from Different Housing Systems. Vet. Rec..

[34] Hoekstra K.E., Nielsen B.D., Orth M.W., Rosenstein D.S., Schott H.C., Shelle J.E. (1999). Comparison of Bone Mineral Content and Biochemical Markers of Bone Metabolism in Stall- vs. Pasture-Reared Horses. Equine Vet. J. Suppl..

[35] Porr C.A., Kronfeld D.S., Lawrence L.A., Pleasant R.S., Harris P.A. (1998). Deconditioning Reduces Mineral Content of the Third Metacarpal Bone in Horses. J. Anim. Sci..

[36] Bell R.A., Nielsen B.D., Waite K., Rosenstein D., Orth M. (2001). Daily Access to Pasture Turnout Prevents Loss of Mineral in the Third Metacarpus of Arabian Weanlings. J. Anim. Sci..

[37] Nielsen B.D., Waite K.L., Bell R.A., Rosenstein D.S., Lindner A. (2000). Long-Term Pasture Housing Promotes Bone Mineral Deposition in the Third Metacarpus of Previously Stalled Weanlings. The Elite Show Jumper.

[38] Logan A.A., Nielsen B.D., Sehl R., Jones E., Robison C.I., Pease A.P. (2019). Short-Term Stall Housing of Horses Results in Changes of Markers of Bone Metabolism. Comp. Exerc. Physiol..

[39] Hiney K.M., Nielsen B.D., Rosenstein D., Orth M.W., Marks B.P. (2004). High-Intensity Exercise of Short Duration Alters Bovine Bone Density and Shape. J. Anim. Sci..

[40] Hiney K.M., Nielsen B.D., Rosenstein D. (2004). Short-Duration Exercise and Confinement Alters Bone Mineral Content and Shape in Weanling Horses. J. Anim. Sci..

[41] Lay D.C., Schenck E.L., Mcmunn K.A., Rosenstein D.S., Stroshine R.L., Nielsen B.D., Richert B.T., Marchant-Forde J.N. (2008). Exercising Stall-Housed Gestating Gilts: Effects on Lameness, the Musculo-Skeletal System, Production, and Behavior. J. Anim. Sci..

[42] Logan A.A., Nielsen B.D., Robison C.I., Manfredi J.M., Buskirk D.D., Schott H.C., Hiney K.M. (2019). Calves, as a Model for Juvenile Horses, Need Only One Sprint per Week to Experience Increased Bone Strength. J. Anim. Sci..

[43] Logan A.A., Nielsen B.D., Hiney K.M., Robison C.I., Manfredi J.M., Buskirk D.D., Popovich J.M. (2022). The Impact of Circular Exercise Diameter on Bone and Joint Health of Juvenile Animals. Animals.

[44] Lanyon L.E. (1984). Functional Strain as a Determinant for Bone Remodeling. Calcif. Tissue Int..

[45] Inman C.L., Warren G.L., Hogan H.A., Bloomfield S.A. (1999). Mechanical Loading Attenuates Bone Loss Due to Immobilization and Calcium Deficiency. J. Appl. Physiol..

[46] Rubin C.T., Lanyon L.E. (1984). Regulation of Bone Formation by Applied Dynamic Loads. J. Bone Jt. Surg..

[47] Nielsen B.D., O’Connor C.I., Rosenstein D.S., Schott H.C., Clayton H.M. (2002). Influence of Trotting and Supplemental Weight on Metacarpal Bone Development. Equine Vet. J. Suppl..

[48] Maderious W.E. (1972). The Bucked Shin Complex. Proc. Amer. Assoc. Equine Pract..

[49] Spooner H.S., Nielsen B.D., Woodward A.D., Rosenstein D.S., Harris P.A. (2008). Endurance Training Has Little Impact on Mineral Content of the Third Metacarpus in Two-Year-Old Arabian Horses. J. Equine Vet. Sci..

[50] Silvers B.L., Leatherwood J.L., Arnold C.E., Nielsen B.D., Huseman C.J., Dominguez B.J., Glass K.G., Martinez R.E., Much M.L., Bradbery A.N. (2020). Effects of Aquatic Conditioning on Cartilage and Bone Metabolism in Young Horses. J. Anim. Sci..

[51] Keller G.A., Nielsen B.D., Vergara-Hernandez F.B., Robison C.I. (2022). Tracking the Impact of Weather on Equine Activity While Pastured. J. Equine Vet. Sci..

[52] Heleski C.R., Shelle A.C., Nielsen B.D., Zanella A.J. (2002). Influence of Housing on Weanling Horse Behavior and Subsequent Welfare. Appl. Anim. Behav. Sci..

[53] Vergara-Hernandez F.B., Nielsen B.D., Colbath A.C. (2022). Is the Use of Bisphosphonates Putting Horses at Risk? An Osteoclast Perspective. Animals.

[54] Pritchard A., Nielsen B.D., Robison C., Spooner H. (2020). Furosemide Administration Results in a Transient Alteration in Calcium Balance in Mature Horses. J. Anim. Physiol. Anim. Nutr..

[55] Nielsen B.D., Eckert S.M., Robison C.I., Mills J., Peters D., Pease A., Schott II H.C. (2017). Omeprazole and Its Impact on Mineral Absorption in Horses. Anim. Prod. Sci..

[56] Logan A.A., Nielsen B.D. (2021). Training Young Horses: The Science behind the Benefits. Animals.

[57] Nielsen B.D., Kawcak C.E., Lindner A. (2010). Considerations of the Optimal Management of Horses from Birth to 2 Years of Age. Performance Diagnosis and Purchase Examination of Elite Sport Horses.

[58] Nielsen B.D., Spooner H.S. (2008). Small Changes in Exercise, Not Nutrition, Often Result in Measurable Changes in Bone. Comp. Exerc. Physiol..

[59] McCulloch R.G., Bailey D.A., Houston C.S., Dodd B.L. (1990). Effects of Physical Activity, Dietary Calcium Intake and Selected Lifestyle Factors on Bone Density in Young Women. Can. Med. Assoc. J..

[60] Borer K.T. (2005). Physical Activity in the Prevention and Amelioration of Osteoporosis in Women. Sport. Med..

[61] Nielsen B.D. (2008). The Other Steroid. Am. Quart. Racing J..

[62] Warden S.J., Edwards W.B., Willy R.W. (2021). Preventing Bone Stress Injuries in Runners with Optimal Workload. Curr. Osteoporos. Rep..

